# 3-(4-Chloro­anilino)-5,5-dimethyl­cyclo­hex-2-en-1-one

**DOI:** 10.1107/S1600536812010495

**Published:** 2012-03-14

**Authors:** Sumati Anthal, Ambika Sambyal, R. K. Bamzai, Rajni Kant, Vivek K. Gupta

**Affiliations:** a Post-Graduate Department of Physics & Electronics, University of Jammu, Jammu Tawi 180 006, India; b Post-Graduate Department of Chemistry, University of Jammu, Jammu Tawi 180 006, India

## Abstract

The asymmetric unit of the title compound, C_14_H_16_ClNO, contains two independent mol­ecules, both with the cyclo­hexene ring in a sofa conformation. In the crystal, N—H⋯O hydrogen bonds link the mol­ecules related by translation along the *a* axis into two crystallographically independent chains. Weak C—H⋯π inter­actions are also observed.

## Related literature

For related structures, see: Bertolasi *et al.* (1998 ▶); Mehdi *et al.* (2010 ▶). For general background to enamines as versatile substrates for the preparation of bioactive alkaloids, see: Heller & Natarajan (2006 ▶); Katritzky *et al.* (1993 ▶); Campaigine & Lake (1959 ▶). For bond-length data, see: Allen *et al.* (1987 ▶).
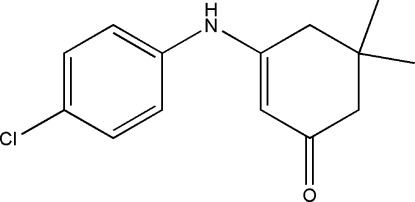


## Experimental

### 

#### Crystal data

C_14_H_16_ClNO
*M* *_r_* = 249.73
Monoclinic, 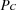

*a* = 7.4103 (2) Å
*b* = 15.1916 (5) Å
*c* = 11.6408 (4) Å
β = 99.443 (3)°
*V* = 1292.70 (7) Å^3^
*Z* = 4
Mo *K*α radiation
μ = 0.28 mm^−1^
*T* = 293 K
0.30 × 0.20 × 0.20 mm

#### Data collection

Oxford Diffraction Xcalibur Sapphire3 diffractometer
Absorption correction: multi-scan (*CrysAlis RED*; Oxford Diffraction, 2010 ▶) *T*_min_ = 0.961, *T*_max_ = 1.000
19792 measured reflections
5570 independent reflections
4069 reflections with *I* > 2σ(*I*)
*R*_int_ = 0.034

#### Refinement

*R*[*F* ^2^ > 2σ(*F* ^2^)] = 0.043
*wR*(*F* ^2^) = 0.100
*S* = 1.03
5570 reflections
319 parameters
2 restraints
H atoms treated by a mixture of independent and constrained refinement
Δρ_max_ = 0.15 e Å^−3^
Δρ_min_ = −0.21 e Å^−3^
Absolute structure: Flack (1983 ▶), 2721 Friedel pairs
Flack parameter: −0.04 (5)

### 

Data collection: *CrysAlis PRO* (Oxford Diffraction, 2010 ▶); cell refinement: *CrysAlis PRO*; data reduction: *CrysAlis PRO*; program(s) used to solve structure: *SHELXS97* (Sheldrick, 2008 ▶); program(s) used to refine structure: *SHELXL97* (Sheldrick, 2008 ▶); molecular graphics: *ORTEP-3* (Farrugia, 1997 ▶); software used to prepare material for publication: *PLATON* (Spek, 2009 ▶).

## Supplementary Material

Crystal structure: contains datablock(s) I, global. DOI: 10.1107/S1600536812010495/cv5258sup1.cif

Structure factors: contains datablock(s) I. DOI: 10.1107/S1600536812010495/cv5258Isup2.hkl

Supplementary material file. DOI: 10.1107/S1600536812010495/cv5258Isup3.cml

Additional supplementary materials:  crystallographic information; 3D view; checkCIF report

## Figures and Tables

**Table 1:** Hydrogen-bond geometry (Å, °) *Cg*1 and *Cg*2 are the centroids of the C9*A*–C14*A* and C9*B*–C14*B* rings, respectively.

*D*—H⋯*A*	*D*—H	H⋯*A*	*D*⋯*A*	*D*—H⋯*A*
N1*A*—H1*A*⋯O1*A*^i^	0.85 (3)	2.02 (3)	2.852 (3)	165 (2)
N1*B*—H1*B*⋯O1*B*^i^	0.83 (3)	2.02 (3)	2.833 (3)	165 (2)
C7*A*—H72*A*⋯*Cg*1^ii^	0.96	2.71	3.637 (3)	163
C8*B*—H83*B*⋯*Cg*2^iii^	0.96	2.70	3.640 (3)	167

Symmetry codes: (i) 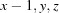; (ii) 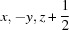; (iii) 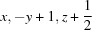.

**Fig. 1.**
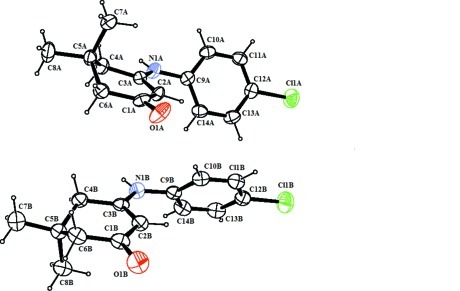
ORTEP view of the molecule with the atom-labeling scheme. The displacement ellipsoids are drawn at the 40% probability level. H atoms are shown as small spheres of arbitrary radii.

## supplementary crystallographic information

### Comment

Enamines are versatile substrates for the preparation of useful bioactive alkaloids, such as pyrazoles (Heller & Natarajan 2006), quinolines (Katritzky *et al.*,1993) and carbazoles (Campaigine & Lake 1959). The compounds are generally prepared by heating aldehydes or ketones with primary amines in presence of strong acids.However, these methods are associated with the limitations of low yield, undesirable side reactions and polymerization. Therefore, there is a need to develop an alternative efficient methodology for the preparation of these compounds, under ambient reaction conditions.Herein, we describe the preparation and XRD studies of the model the title compound from dimedone and *p*-chloroaniline in the presence of triethylammonium trifluoromethanesulfonate and triethylamine at room temperature.

The asymmetric unit of the title compound comprises of two crystallographically independent molecules, A and B, respectively (Fig. 1). The geometry of both the asymmetric molecules, A and B, indicates a high degree of similarity in terms of their bond distances and bond angles. A comparison of these parameters with some related structures (Mehdi *et al.*, 2010; Bertolasi *et al.*, 1998) indicates a good agreement. The average aromatic bond length in the phenyl ring is 1.381 (3) Å for molecule A and 1.380 (3) Å for molecule B. For both the molecules, the average observed bond angle in the phenyl ring is 120.0 ° which coincides exactly with the theoretical value of sp2 hybridization. The length of the double bond C1=O1 [1.244 (3) (molecule A) and 1.247 (3) Å (molecule B)] is larger than the standard value for carbonyl group [1.192 Å] (Allen *et al.*, 1987)and lengthening of the C1=O1 double bond is due to strong intermolecular hydrogen bond between N1 and O1. The dihedral angle between the cyclohexene ring and phenyl ring is 58.2 (1)° (molecule A) and 57.5 (1)° (molecule B). In cyclohexene ring, the C2=C3 distance of 1.361 (3) Å (molecule A) and 1.370 (3) Å (molecule B) confirms the localization of a double bond at this position. This double bond imposes a sofa conformation on cyclohexene ring, with asymmetry parameters: ΔCs (C2A—C5A) (molecule A) = 6.11; ΔCs (C2B—C5B) (molecule B) = 3.30.

In the crystal, adjacent molecules are interconnected through N—H···O hydrogen bonds (Table 1). The crystal structure is further stabilized by C—H···π hydrogen bonding (Table 1, *Cg*1 and *Cg*2 represent the centre of gravity of benzene ring C9—C14 in molecules A and B, respectively).

### Experimental

Dimedone (1 *x* 10–3 mol) and 4-chloroaniline (1 *x* 10–3 mol) were taken in dry methanol (50 ml). To this mixture were added triethyl ammonium trifluoromethanesulfonate (30 mol %) and triethylamine (1 mol. equiv.). The reaction was refluxed on water bath. The reaction was monitored by thin layer chromatography, using methylene chloride - ethyl acetate (19: 1v/v) as solvent system. On completion of reaction (6 h) the contents of the flask were triturated with water and extracted with ethyl acetate. The organic layer was washed successively with brine (2 *x* 25 ml) and water (4 *x* 30 ml) dried on anhydrous sodium sulfate and filtered. The solvent was removed under reduced pressure. The residue was crystallized from chloroformmethanol(1: 25 *v*/*v*) to give compound 1, in 95% yield. For XRD studies, title compound was further purified by column chromatography on silica gel and crystallized again from chloroform-methanol. Single crystals were prepared by slow evaporation of its solution in chloroform. The structure of the compound was ascertained by spectral methods (MS, IR,1*H*-NMR, 13 C NMR, DEPT 135°). IR (KBr):νmaxcm-1 3451, 3250, 2922, 1635, 1597, 1565, 1493, 1369, 1242, 1171, 1088,1015, 1000, 924. 1*H*-NMR (400 MHz, CDCl3):δCH3x2), 2.19 (s, 2H, Hax2), 1.74 (s br exch. D2O, NHx1), 2.33 (s, 2H, Hex2), 5.49 (s, 1H, H-2), 7.07 (d, J= 8.8 Hz, 2H, H-arom), 7.27(d, J= 8.8 Hz, 2H). 13CNMR(100 MHz,CDCl3):δc 21.29, 28.2, 43.2, 50.2, 98.5, 124.9, 125.8, 128.7, 129.6, 130.6, 136.9, 160.7, 198.3.MS m/*z* 251.0870 (76), 249.0845 (*M*+) (100) (calc. for C14H16ClNO 249.0837), 233 (52), 231 (75), 94 (76), 81 (73).

### Refinement

H1A attached to N1A and H1B attached to N1B were located from the difference map and isotropically refined with the restraints N—H = 0.84 (3) Å. The remaining H atoms were positioned geometrically and were treated as riding on their parent C atoms, with C—H distances of 0.93–0.97 Å; and with *U*_iso_(H) = 1.2-1.5 U_eq_(C).

### Crystal data

**Table:**

C_14_H_16_ClNO	*F*(000) = 528
*M**_r_* = 249.73	*D*_x_ = 1.283 Mg m^−^^3^
Monoclinic, *P**c*	Mo *K*α radiation, λ = 0.71073 Å
Hall symbol: P -2yc	Cell parameters from 7292 reflections
*a* = 7.4103 (2) Å	θ = 3.5–29.1°
*b* = 15.1916 (5) Å	µ = 0.28 mm^−^^1^
*c* = 11.6408 (4) Å	*T* = 293 K
β = 99.443 (3)°	Block, white
*V* = 1292.70 (7) Å^3^	0.30 × 0.20 × 0.20 mm
*Z* = 4	

### Data collection

**Table:**

Oxford Diffraction Xcalibur Sapphire3 diffractometer	5570 independent reflections
Radiation source: fine-focus sealed tube	4069 reflections with *I* > 2σ(*I*)
Graphite monochromator	*R*_int_ = 0.034
Detector resolution: 0 pixels mm^-1^	θ_max_ = 27.0°, θ_min_ = 3.6°
ω scans	*h* = −9→9
Absorption correction: multi-scan (*CrysAlis RED*; Oxford Diffraction, 2010)	*k* = −19→19
*T*_min_ = 0.961, *T*_max_ = 1.000	*l* = −14→14
19792 measured reflections	

### Refinement

**Table:**

Refinement on *F*^2^	Secondary atom site location: difference Fourier map
Least-squares matrix: full	Hydrogen site location: inferred from neighbouring sites
*R*[*F*^2^ > 2σ(*F*^2^)] = 0.043	H atoms treated by a mixture of independent and constrained refinement
*wR*(*F*^2^) = 0.100	*w* = 1/[σ^2^(*F*_o_^2^) + (0.0374*P*)^2^ + 0.1217*P*] where *P* = (*F*_o_^2^ + 2*F*_c_^2^)/3
*S* = 1.03	(Δ/σ)_max_ = 0.001
5570 reflections	Δρ_max_ = 0.15 e Å^−^^3^
319 parameters	Δρ_min_ = −0.21 e Å^−^^3^
2 restraints	Absolute structure: Flack (1983), 2721 Friedel pairs
Primary atom site location: structure-invariant direct methods	Flack parameter: −0.04 (5)

### Special details

**Table:**

Experimental. *CrysAlis PRO*, Oxford Diffraction Ltd., Version 1.171.34.40 (release 27–08-2010 CrysAlis171. NET) (compiled Aug 27 2010,11:50:40) Empirical absorption correction using spherical harmonics, implemented in SCALE3 ABSPACK scaling algorithm.
Geometry. All e.s.d.'s (except the e.s.d. in the dihedral angle between two l.s. planes) are estimated using the full covariance matrix. The cell e.s.d.'s are taken into account individually in the estimation of e.s.d.'s in distances, angles and torsion angles; correlations between e.s.d.'s in cell parameters are only used when they are defined by crystal symmetry. An approximate (isotropic) treatment of cell e.s.d.'s is used for estimating e.s.d.'s involving l.s. planes.
Refinement. Refinement of *F*^2^ against ALL reflections. The weighted *R*-factor *wR* and goodness of fit *S* are based on *F*^2^, conventional *R*-factors *R* are based on *F*, with *F* set to zero for negative *F*^2^. The threshold expression of *F*^2^ > σ(*F*^2^) is used only for calculating *R*-factors(gt) *etc*. and is not relevant to the choice of reflections for refinement. *R*-factors based on *F*^2^ are statistically about twice as large as those based on *F*, and *R*- factors based on ALL data will be even larger.

### Fractional atomic coordinates and isotropic or equivalent isotropic displacement parameters (Å^2^)

**Table:**

	*x*	*y*	*z*	*U*_iso_*/*U*_eq_	
Cl1A	0.02376 (10)	0.84221 (5)	−0.37777 (6)	0.0695 (3)	
N1A	0.1625 (3)	0.86014 (14)	0.13741 (18)	0.0394 (5)	
C1A	0.6616 (3)	0.87416 (14)	0.2364 (2)	0.0361 (6)	
C3A	0.3290 (3)	0.86484 (13)	0.20386 (19)	0.0307 (5)	
O1A	0.8071 (2)	0.88210 (13)	0.19728 (17)	0.0576 (6)	
C6A	0.6634 (3)	0.87541 (16)	0.3650 (2)	0.0418 (6)	
H61A	0.7716	0.9066	0.4022	0.050*	
H62A	0.6712	0.8154	0.3938	0.050*	
C4A	0.3259 (3)	0.87268 (15)	0.3326 (2)	0.0400 (6)	
H42A	0.3182	0.8142	0.3649	0.048*	
H41A	0.2170	0.9048	0.3440	0.048*	
C12A	0.0607 (3)	0.84585 (15)	−0.2259 (2)	0.0404 (6)	
C13A	0.1651 (3)	0.78103 (16)	−0.1636 (2)	0.0441 (7)	
H13	0.2131	0.7353	−0.2022	0.053*	
C2A	0.4897 (3)	0.86468 (14)	0.1616 (2)	0.0322 (5)	
H2A	0.4875	0.8582	0.0820	0.039*	
C10A	0.0213 (3)	0.91674 (15)	−0.0498 (2)	0.0422 (6)	
H10A	−0.0282	0.9621	−0.0111	0.051*	
C14A	0.1978 (3)	0.78474 (15)	−0.0433 (2)	0.0399 (6)	
H14A	0.2665	0.7410	−0.0007	0.048*	
C11A	−0.0134 (3)	0.91349 (15)	−0.1705 (2)	0.0431 (6)	
H11A	−0.0853	0.9562	−0.2130	0.052*	
C5A	0.4937 (3)	0.91935 (15)	0.39910 (19)	0.0366 (6)	
C9A	0.1283 (3)	0.85343 (14)	0.0136 (2)	0.0340 (6)	
C7A	0.4922 (3)	1.01738 (15)	0.3674 (2)	0.0436 (6)	
H72A	0.3848	1.0447	0.3876	0.065*	
H73A	0.4916	1.0236	0.2853	0.065*	
H71A	0.5993	1.0453	0.4095	0.065*	
C8A	0.4948 (4)	0.9102 (2)	0.5304 (2)	0.0578 (8)	
H83A	0.4911	0.8490	0.5503	0.087*	
H81A	0.3899	0.9396	0.5509	0.087*	
H82A	0.6042	0.9364	0.5721	0.087*	
Cl1B	0.53853 (10)	0.65806 (5)	−0.33380 (7)	0.0718 (3)	
C1B	1.1802 (3)	0.62985 (15)	0.2807 (2)	0.0398 (6)	
C11B	0.6804 (3)	0.71986 (15)	−0.1197 (2)	0.0431 (6)	
H11B	0.7258	0.7663	−0.1585	0.052*	
C12B	0.5780 (3)	0.65421 (16)	−0.1819 (2)	0.0429 (6)	
C3B	0.8480 (3)	0.63611 (14)	0.2483 (2)	0.0342 (6)	
N1B	0.6810 (3)	0.64009 (13)	0.1810 (2)	0.0416 (6)	
C4B	0.8453 (3)	0.62493 (15)	0.3765 (2)	0.0395 (6)	
H42B	0.7358	0.5929	0.3866	0.047*	
H41B	0.8391	0.6826	0.4116	0.047*	
C2B	1.0094 (3)	0.64015 (14)	0.2057 (2)	0.0376 (6)	
H2B	1.0074	0.6497	0.1266	0.045*	
C6B	1.1831 (3)	0.62237 (15)	0.4087 (2)	0.0420 (6)	
H62B	1.1919	0.6809	0.4424	0.050*	
H61B	1.2914	0.5899	0.4428	0.050*	
C14B	0.5403 (3)	0.58314 (15)	−0.0062 (2)	0.0448 (7)	
H14B	0.4913	0.5376	0.0323	0.054*	
C10B	0.7145 (3)	0.71586 (15)	−0.0002 (2)	0.0397 (6)	
H10B	0.7831	0.7598	0.0422	0.048*	
C9B	0.6466 (3)	0.64625 (15)	0.0577 (2)	0.0351 (6)	
C13B	0.5053 (3)	0.58639 (16)	−0.1263 (2)	0.0480 (7)	
H13'	0.4337	0.5434	−0.1688	0.058*	
O1B	1.3270 (2)	0.62702 (14)	0.24154 (19)	0.0645 (6)	
C5B	1.0126 (3)	0.57602 (14)	0.4406 (2)	0.0374 (6)	
C7B	1.0128 (4)	0.58084 (17)	0.5718 (2)	0.0543 (7)	
H73B	0.9032	0.5541	0.5896	0.081*	
H72B	1.0177	0.6413	0.5961	0.081*	
H71B	1.1175	0.5501	0.6122	0.081*	
C8B	1.0106 (3)	0.47940 (15)	0.4026 (2)	0.0467 (7)	
H83B	0.9045	0.4508	0.4225	0.070*	
H81B	1.1189	0.4505	0.4414	0.070*	
H82B	1.0072	0.4763	0.3199	0.070*	
H1B	0.587 (4)	0.6320 (16)	0.210 (2)	0.057 (8)*	
H1A	0.067 (4)	0.8708 (15)	0.167 (2)	0.047 (7)*	

### Atomic displacement parameters (Å^2^)

**Table:**

	*U*^11^	*U*^22^	*U*^33^	*U*^12^	*U*^13^	*U*^23^
Cl1A	0.0762 (7)	0.0863 (6)	0.0423 (6)	0.0071 (4)	−0.0012 (7)	−0.0017 (4)
N1A	0.0234 (11)	0.0560 (13)	0.0409 (14)	−0.0008 (8)	0.0113 (10)	−0.0050 (9)
C1A	0.0256 (13)	0.0372 (13)	0.0465 (16)	0.0029 (9)	0.0089 (11)	−0.0111 (10)
C3A	0.0266 (12)	0.0317 (11)	0.0347 (14)	−0.0023 (9)	0.0074 (11)	−0.0006 (9)
O1A	0.0259 (10)	0.0883 (14)	0.0613 (13)	−0.0067 (9)	0.0147 (9)	−0.0300 (10)
C6A	0.0349 (14)	0.0495 (15)	0.0380 (15)	0.0096 (11)	−0.0029 (12)	−0.0020 (11)
C4A	0.0364 (14)	0.0477 (14)	0.0394 (15)	−0.0050 (11)	0.0173 (12)	0.0029 (11)
C12A	0.0329 (13)	0.0518 (14)	0.0352 (15)	−0.0047 (10)	0.0017 (11)	−0.0027 (11)
C13A	0.0347 (14)	0.0463 (14)	0.0489 (18)	0.0058 (10)	−0.0005 (13)	−0.0120 (11)
C2A	0.0254 (12)	0.0430 (13)	0.0299 (13)	0.0000 (9)	0.0096 (10)	−0.0046 (10)
C10A	0.0315 (14)	0.0429 (14)	0.0533 (18)	0.0042 (11)	0.0104 (12)	−0.0051 (12)
C14A	0.0305 (13)	0.0387 (13)	0.0480 (17)	0.0047 (10)	−0.0004 (12)	−0.0019 (11)
C11A	0.0356 (14)	0.0414 (14)	0.0509 (18)	0.0066 (10)	0.0034 (13)	0.0041 (11)
C5A	0.0370 (14)	0.0443 (13)	0.0297 (14)	0.0021 (10)	0.0091 (12)	0.0028 (10)
C9A	0.0194 (11)	0.0412 (13)	0.0418 (16)	−0.0026 (9)	0.0062 (11)	−0.0016 (10)
C7A	0.0457 (15)	0.0436 (14)	0.0430 (15)	0.0041 (11)	0.0117 (12)	−0.0065 (11)
C8A	0.0677 (19)	0.0769 (19)	0.0302 (15)	0.0024 (15)	0.0121 (14)	0.0026 (13)
Cl1B	0.0730 (7)	0.0900 (7)	0.0497 (7)	−0.0030 (4)	0.0022 (7)	−0.0010 (4)
C1B	0.0206 (12)	0.0401 (14)	0.0596 (19)	−0.0018 (9)	0.0088 (12)	0.0049 (11)
C11B	0.0362 (14)	0.0375 (13)	0.0562 (19)	−0.0032 (10)	0.0097 (13)	0.0027 (12)
C12B	0.0318 (13)	0.0513 (15)	0.0445 (16)	0.0063 (11)	0.0030 (12)	0.0011 (11)
C3B	0.0225 (12)	0.0338 (12)	0.0472 (17)	0.0035 (9)	0.0080 (11)	0.0011 (10)
N1B	0.0189 (11)	0.0573 (13)	0.0504 (15)	−0.0005 (9)	0.0114 (11)	0.0061 (10)
C4B	0.0274 (13)	0.0462 (14)	0.0473 (16)	0.0048 (10)	0.0128 (12)	0.0029 (11)
C2B	0.0241 (13)	0.0433 (13)	0.0464 (16)	0.0008 (9)	0.0089 (12)	0.0061 (11)
C6B	0.0278 (13)	0.0411 (13)	0.0546 (18)	−0.0048 (10)	−0.0003 (12)	0.0032 (11)
C14B	0.0284 (13)	0.0435 (15)	0.062 (2)	−0.0059 (10)	0.0055 (13)	0.0062 (12)
C10B	0.0282 (12)	0.0382 (13)	0.0532 (17)	−0.0041 (9)	0.0082 (12)	−0.0037 (11)
C9B	0.0188 (11)	0.0391 (12)	0.0479 (17)	0.0027 (9)	0.0073 (12)	0.0027 (10)
C13B	0.0324 (14)	0.0439 (15)	0.065 (2)	−0.0054 (11)	−0.0008 (13)	−0.0058 (13)
O1B	0.0222 (10)	0.0935 (15)	0.0808 (17)	0.0033 (9)	0.0176 (11)	0.0169 (11)
C5B	0.0312 (13)	0.0379 (13)	0.0435 (16)	−0.0014 (10)	0.0071 (12)	0.0027 (10)
C7B	0.0528 (17)	0.0598 (17)	0.0499 (18)	−0.0035 (13)	0.0075 (14)	0.0030 (13)
C8B	0.0389 (14)	0.0401 (14)	0.0627 (19)	−0.0020 (11)	0.0127 (13)	0.0049 (12)

### Geometric parameters (Å, º)

**Table:**

Cl1A—C12A	1.744 (3)	Cl1B—C12B	1.745 (3)
N1A—C3A	1.346 (3)	C1B—O1B	1.247 (3)
N1A—C9A	1.426 (3)	C1B—C2B	1.424 (3)
N1A—H1A	0.85 (3)	C1B—C6B	1.491 (4)
C1A—O1A	1.244 (3)	C11B—C10B	1.374 (3)
C1A—C2A	1.427 (3)	C11B—C12B	1.383 (3)
C1A—C6A	1.495 (3)	C11B—H11B	0.9300
C3A—C2A	1.361 (3)	C12B—C13B	1.373 (3)
C3A—C4A	1.508 (3)	C3B—N1B	1.353 (3)
C6A—C5A	1.533 (3)	C3B—C2B	1.370 (3)
C6A—H61A	0.9700	C3B—C4B	1.506 (3)
C6A—H62A	0.9700	N1B—C9B	1.418 (3)
C4A—C5A	1.527 (3)	N1B—H1B	0.83 (3)
C4A—H42A	0.9700	C4B—C5B	1.530 (3)
C4A—H41A	0.9700	C4B—H42B	0.9700
C12A—C11A	1.375 (3)	C4B—H41B	0.9700
C12A—C13A	1.382 (3)	C2B—H2B	0.9300
C13A—C14A	1.382 (3)	C6B—C5B	1.544 (3)
C13A—H13	0.9300	C6B—H62B	0.9700
C2A—H2A	0.9300	C6B—H61B	0.9700
C10A—C9A	1.381 (3)	C14B—C9B	1.379 (3)
C10A—C11A	1.387 (3)	C14B—C13B	1.380 (3)
C10A—H10A	0.9300	C14B—H14B	0.9300
C14A—C9A	1.380 (3)	C10B—C9B	1.392 (3)
C14A—H14A	0.9300	C10B—H10B	0.9300
C11A—H11A	0.9300	C13B—H13'	0.9300
C5A—C8A	1.533 (3)	C5B—C7B	1.529 (4)
C5A—C7A	1.534 (3)	C5B—C8B	1.533 (3)
C7A—H72A	0.9600	C7B—H73B	0.9600
C7A—H73A	0.9600	C7B—H72B	0.9600
C7A—H71A	0.9600	C7B—H71B	0.9600
C8A—H83A	0.9600	C8B—H83B	0.9600
C8A—H81A	0.9600	C8B—H81B	0.9600
C8A—H82A	0.9600	C8B—H82B	0.9600
			
C3A—N1A—C9A	125.35 (19)	O1B—C1B—C2B	121.4 (3)
C3A—N1A—H1A	120.2 (18)	O1B—C1B—C6B	119.5 (2)
C9A—N1A—H1A	113.6 (18)	C2B—C1B—C6B	119.1 (2)
O1A—C1A—C2A	121.8 (2)	C10B—C11B—C12B	119.5 (2)
O1A—C1A—C6A	120.0 (2)	C10B—C11B—H11B	120.3
C2A—C1A—C6A	118.2 (2)	C12B—C11B—H11B	120.3
N1A—C3A—C2A	124.5 (2)	C13B—C12B—C11B	121.2 (3)
N1A—C3A—C4A	114.42 (19)	C13B—C12B—Cl1B	119.3 (2)
C2A—C3A—C4A	121.1 (2)	C11B—C12B—Cl1B	119.5 (2)
C1A—C6A—C5A	113.10 (17)	N1B—C3B—C2B	123.9 (2)
C1A—C6A—H61A	109.0	N1B—C3B—C4B	114.8 (2)
C5A—C6A—H61A	109.0	C2B—C3B—C4B	121.2 (2)
C1A—C6A—H62A	109.0	C3B—N1B—C9B	125.8 (2)
C5A—C6A—H62A	109.0	C3B—N1B—H1B	121 (2)
H61A—C6A—H62A	107.8	C9B—N1B—H1B	113 (2)
C3A—C4A—C5A	113.37 (18)	C3B—C4B—C5B	113.35 (19)
C3A—C4A—H42A	108.9	C3B—C4B—H42B	108.9
C5A—C4A—H42A	108.9	C5B—C4B—H42B	108.9
C3A—C4A—H41A	108.9	C3B—C4B—H41B	108.9
C5A—C4A—H41A	108.9	C5B—C4B—H41B	108.9
H42A—C4A—H41A	107.7	H42B—C4B—H41B	107.7
C11A—C12A—C13A	121.2 (2)	C3B—C2B—C1B	121.0 (2)
C11A—C12A—Cl1A	119.41 (19)	C3B—C2B—H2B	119.5
C13A—C12A—Cl1A	119.40 (19)	C1B—C2B—H2B	119.5
C14A—C13A—C12A	119.5 (2)	C1B—C6B—C5B	113.41 (19)
C14A—C13A—H13	120.2	C1B—C6B—H62B	108.9
C12A—C13A—H13	120.2	C5B—C6B—H62B	108.9
C3A—C2A—C1A	121.7 (2)	C1B—C6B—H61B	108.9
C3A—C2A—H2A	119.1	C5B—C6B—H61B	108.9
C1A—C2A—H2A	119.1	H62B—C6B—H61B	107.7
C9A—C10A—C11A	120.7 (2)	C9B—C14B—C13B	121.2 (2)
C9A—C10A—H10A	119.6	C9B—C14B—H14B	119.4
C11A—C10A—H10A	119.6	C13B—C14B—H14B	119.4
C9A—C14A—C13A	120.0 (2)	C11B—C10B—C9B	120.2 (2)
C9A—C14A—H14A	120.0	C11B—C10B—H10B	119.9
C13A—C14A—H14A	120.0	C9B—C10B—H10B	119.9
C12A—C11A—C10A	118.7 (2)	C14B—C9B—C10B	119.1 (2)
C12A—C11A—H11A	120.6	C14B—C9B—N1B	119.5 (2)
C10A—C11A—H11A	120.6	C10B—C9B—N1B	121.4 (2)
C4A—C5A—C6A	107.51 (19)	C12B—C13B—C14B	118.8 (2)
C4A—C5A—C8A	109.5 (2)	C12B—C13B—H13'	120.6
C6A—C5A—C8A	110.39 (19)	C14B—C13B—H13'	120.6
C4A—C5A—C7A	110.86 (19)	C7B—C5B—C4B	109.3 (2)
C6A—C5A—C7A	109.52 (19)	C7B—C5B—C8B	109.45 (19)
C8A—C5A—C7A	109.0 (2)	C4B—C5B—C8B	110.84 (19)
C14A—C9A—C10A	119.8 (2)	C7B—C5B—C6B	110.6 (2)
C14A—C9A—N1A	121.4 (2)	C4B—C5B—C6B	106.90 (18)
C10A—C9A—N1A	118.8 (2)	C8B—C5B—C6B	109.67 (19)
C5A—C7A—H72A	109.5	C5B—C7B—H73B	109.5
C5A—C7A—H73A	109.5	C5B—C7B—H72B	109.5
H72A—C7A—H73A	109.5	H73B—C7B—H72B	109.5
C5A—C7A—H71A	109.5	C5B—C7B—H71B	109.5
H72A—C7A—H71A	109.5	H73B—C7B—H71B	109.5
H73A—C7A—H71A	109.5	H72B—C7B—H71B	109.5
C5A—C8A—H83A	109.5	C5B—C8B—H83B	109.5
C5A—C8A—H81A	109.5	C5B—C8B—H81B	109.5
H83A—C8A—H81A	109.5	H83B—C8B—H81B	109.5
C5A—C8A—H82A	109.5	C5B—C8B—H82B	109.5
H83A—C8A—H82A	109.5	H83B—C8B—H82B	109.5
H81A—C8A—H82A	109.5	H81B—C8B—H82B	109.5
			
C9A—N1A—C3A—C2A	0.7 (4)	C10B—C11B—C12B—C13B	1.9 (4)
C9A—N1A—C3A—C4A	179.28 (19)	C10B—C11B—C12B—Cl1B	−178.72 (17)
O1A—C1A—C6A—C5A	145.2 (2)	C2B—C3B—N1B—C9B	2.0 (4)
C2A—C1A—C6A—C5A	−34.1 (3)	C4B—C3B—N1B—C9B	−176.87 (19)
N1A—C3A—C4A—C5A	−153.0 (2)	N1B—C3B—C4B—C5B	150.9 (2)
C2A—C3A—C4A—C5A	25.6 (3)	C2B—C3B—C4B—C5B	−28.0 (3)
C11A—C12A—C13A—C14A	−0.8 (4)	N1B—C3B—C2B—C1B	−175.9 (2)
Cl1A—C12A—C13A—C14A	178.69 (18)	C4B—C3B—C2B—C1B	2.9 (3)
N1A—C3A—C2A—C1A	176.9 (2)	O1B—C1B—C2B—C3B	174.7 (2)
C4A—C3A—C2A—C1A	−1.6 (3)	C6B—C1B—C2B—C3B	−5.3 (3)
O1A—C1A—C2A—C3A	−173.3 (2)	O1B—C1B—C6B—C5B	−147.5 (2)
C6A—C1A—C2A—C3A	6.0 (3)	C2B—C1B—C6B—C5B	32.5 (3)
C12A—C13A—C14A—C9A	−0.8 (3)	C12B—C11B—C10B—C9B	0.1 (3)
C13A—C12A—C11A—C10A	1.2 (4)	C13B—C14B—C9B—C10B	2.1 (3)
Cl1A—C12A—C11A—C10A	−178.33 (17)	C13B—C14B—C9B—N1B	−179.3 (2)
C9A—C10A—C11A—C12A	0.1 (3)	C11B—C10B—C9B—C14B	−2.0 (3)
C3A—C4A—C5A—C6A	−50.4 (2)	C11B—C10B—C9B—N1B	179.46 (19)
C3A—C4A—C5A—C8A	−170.3 (2)	C3B—N1B—C9B—C14B	122.7 (2)
C3A—C4A—C5A—C7A	69.3 (2)	C3B—N1B—C9B—C10B	−58.8 (3)
C1A—C6A—C5A—C4A	54.9 (2)	C11B—C12B—C13B—C14B	−1.8 (4)
C1A—C6A—C5A—C8A	174.3 (2)	Cl1B—C12B—C13B—C14B	178.80 (18)
C1A—C6A—C5A—C7A	−65.6 (2)	C9B—C14B—C13B—C12B	−0.2 (4)
C13A—C14A—C9A—C10A	2.1 (3)	C3B—C4B—C5B—C7B	171.04 (19)
C13A—C14A—C9A—N1A	−178.7 (2)	C3B—C4B—C5B—C8B	−68.2 (3)
C11A—C10A—C9A—C14A	−1.8 (3)	C3B—C4B—C5B—C6B	51.3 (3)
C11A—C10A—C9A—N1A	179.1 (2)	C1B—C6B—C5B—C7B	−172.9 (2)
C3A—N1A—C9A—C14A	58.4 (3)	C1B—C6B—C5B—C4B	−53.9 (2)
C3A—N1A—C9A—C10A	−122.4 (2)		

### Hydrogen-bond geometry (Å, º)

Cg1 and Cg2 are the centroids of the C9A–C14A and C9B–C14B rings, respectively.

**Table:**

*D*—H···*A*	*D*—H	H···*A*	*D*···*A*	*D*—H···*A*
N1*A*—H1*A*···O1*A*^i^	0.85 (3)	2.02 (3)	2.852 (3)	165 (2)
N1*B*—H1*B*···O1*B*^i^	0.83 (3)	2.02 (3)	2.833 (3)	165 (2)
C7*A*—H72*A*···*Cg*1^ii^	0.96	2.71	3.637 (3)	163
C8*B*—H83*B*···*Cg*2^iii^	0.96	2.70	3.640 (3)	167

Symmetry codes: (i) *x*−1, *y*, *z*; (ii) *x*, −*y*, *z*+1/2; (iii) *x*, −*y*+1, *z*+1/2.
